# Liquid biopsy in orthopedic trauma: exosomes as functional mediators and mechanistic indicators in post-traumatic complications

**DOI:** 10.3389/fmed.2025.1707928

**Published:** 2025-11-05

**Authors:** Jinyu Gan, Shuangting Zhong, Juan Xie, Liqun Zou

**Affiliations:** ^1^Department of Emergency Medicine, West China Hospital, Sichuan University, Chengdu, China; ^2^West China School of Nursing, Sichuan University, Chengdu, China; ^3^Disaster Medical Center, Sichuan University, Chengdu, China; ^4^Nursing Key Laboratory of Sichuan Province, Chengdu, China

**Keywords:** exosomes, orthopedic trauma, intercellular communication, ischemia–reperfusion, endothelial dysfunction, hypercoagulability, bone regeneration

## Abstract

Severe orthopedic trauma initiates complex pathophysiological cascades that frequently lead to life-threatening complications including acute compartment syndrome, fat embolism syndrome, deep vein thrombosis, and fracture non-union. Traditional biomarkers provide only retrospective indicators of tissue damage, lacking the sensitivity and specificity needed for early complication detection. Exosomes, nanoscale extracellular vesicles carrying proteins, lipids, and nucleic acids, have emerged as critical mediators of intercellular communication that actively participate in trauma pathophysiology. This comprehensive review synthesizes accumulating evidence suggesting that exosomes may function as active mediators rather than passive biomarkers in orthopedic trauma complications. Studies demonstrate that trauma-derived exosomes transfer functional cargo including miR-155 and inflammatory proteins that reprogram recipient cell phenotypes and induce endothelial dysfunction. *In vivo* animal models show that mesenchymal stem cell-derived exosomes significantly enhance fracture healing in non-union models. Based on these findings, we present mechanistic models illustrating how trauma-induced exosomes may drive disease progression in each major complication through distinct molecular pathways. Furthermore, we discuss the diagnostic potential of exosomal biomarkers, address current methodological challenges, and outline future research directions for clinical translation. The integration of exosome-based approaches into trauma care represents a paradigm shift that could enable early detection of complications and development of targeted therapeutic interventions, ultimately improving patient outcomes through precision medicine.

## 1 Introduction

Severe orthopedic trauma represents far more than a simple structural failure of the skeletal system; it initiates a complex and often devastating cascade of local and systemic cellular events. Beyond the initial mechanical injury, the affected tissues and the body as a whole are plunged into a state of profound physiological distress, characterized by localized hypoxia, rampant sterile inflammation, and extensive metabolic reprogramming within a host of cell types, including myocytes, endothelial cells, and adipocytes (1, 2). These cellular responses involve activation of key signaling pathways, including hypoxia-inducible factor-1α (HIF-1α) cascades, nuclear factor-κB (NF-κB)-mediated inflammatory responses, and disruption of tissue homeostasis networks. This secondary wave of biological responses is not merely a byproduct of the injury but rather a critical determinant of patient outcomes. The ensuing cellular crosstalk and systemic feedback loops are directly responsible for the development of life-threatening complications such as acute compartment syndrome (ACS), fat embolism syndrome (FES), deep vein thrombosis (DVT), and fracture non-union, which collectively account for the high rates of morbidity and mortality associated with these injuries (3).

For decades, clinicians have relied on a panel of conventional serological biomarkers, such as creatine kinase (CK), lactate dehydrogenase (LDH), and D-dimer, to gauge the severity of trauma and monitor for complications. However, these markers possess fundamental mechanistic shortcomings that limit their utility for early prognostication. CK and LDH are general indicators of tissue necrosis; their elevation signifies that substantial muscle damage has already occurred, making them lagging indicators rather than predictive tools (4). Similarly, D-dimer, a product of fibrin degradation, is notoriously non-specific in the context of polytrauma, as nearly all significant injuries trigger a systemic coagulation and fibrinolysis response. This renders it incapable of distinguishing between a contained systemic reaction and the onset of a specific pathological event like DVT, leading to a high rate of false positives (5, 6). More critically, these traditional markers fail to capture the dynamic intercellular communication networks that orchestrate tissue responses to trauma, providing no insight into the molecular crosstalk between different cell populations or the temporal evolution of signaling cascades that determine tissue fate.

**Figure 1:**
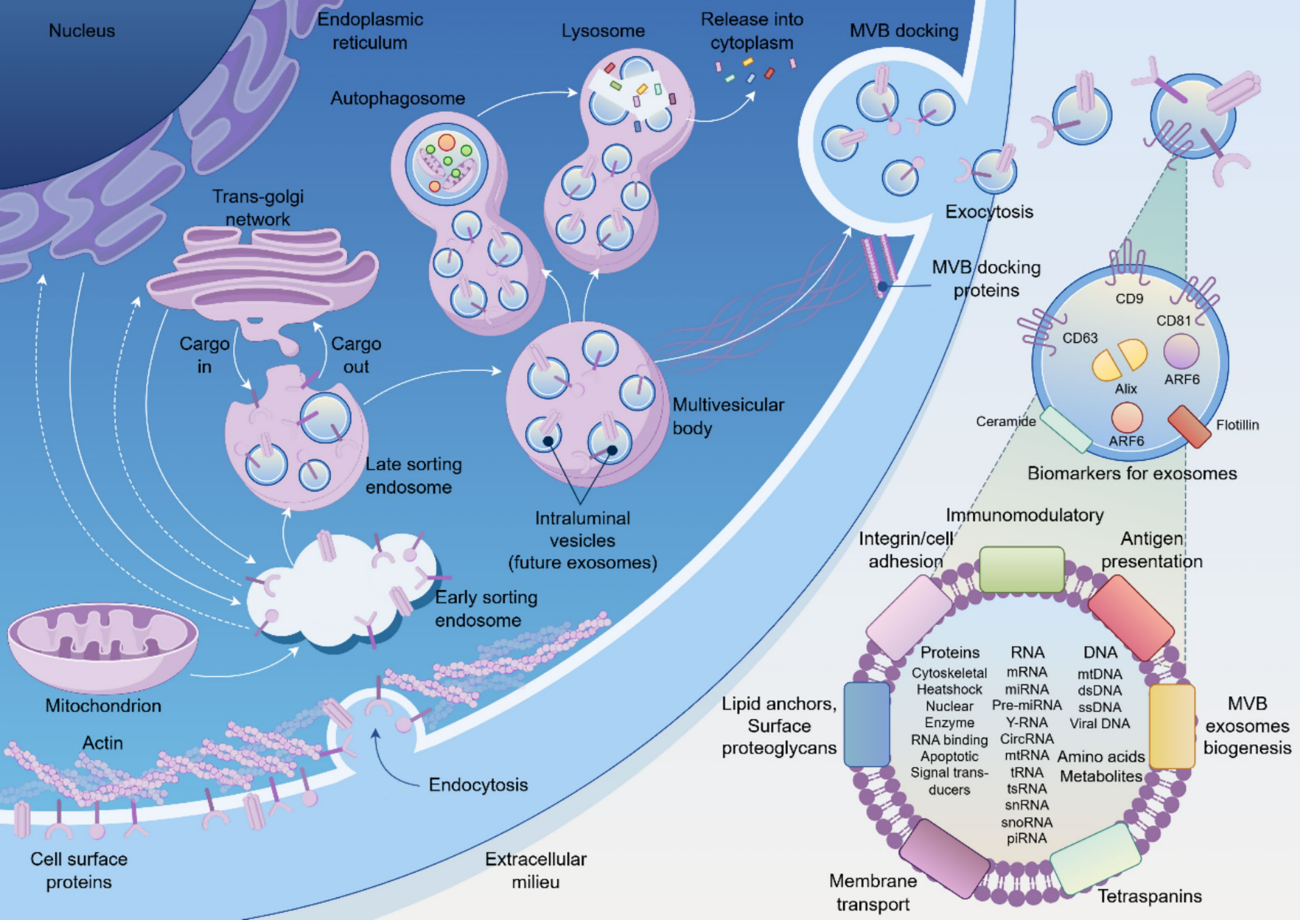
The biogenesis, secretion, and molecular composition of exosomes. Schematic illustration of exosome biogenesis pathway showing the progression from endoplasmic reticulum through the endosomal sorting system to multivesicular body (MVB) formation and subsequent exocytosis. Exosomes contain diverse molecular cargo including membrane proteins (CD9, CD63, CD81, Alix, ARF6), nucleic acids (DNA, RNA species), and metabolites. The released exosomes mediate intercellular communication through various mechanisms including receptor binding, membrane fusion, and cargo transfer to target cells.

In the search for more dynamic and mechanistically informative indicators, exosomes have emerged as a paradigm-shifting class of biomarkers. Exosomes are nanoscale (30–160 nm) extracellular vesicles actively secreted by virtually all cell types as sophisticated vectors for intercellular communication (7). Encased within a stable lipid bilayer, they carry a rich cargo of proteins, lipids, and nucleic acids (including mRNA and miRNA) that provides a molecular snapshot of the originating cell’s real-time physiological or pathological state (8). The biogenesis of exosomes through the endosomal sorting complex required for transport (ESCRT) machinery ensures that their molecular cargo reflects the precise metabolic and transcriptional state of parent cells. Under stress conditions such as hypoxia or inflammation, cells actively remodel their exosomal cargo, enriching for specific microRNAs, metabolic enzymes, and signaling proteins that can reprogram recipient cell behavior (9). This intrinsic ability to transfer functional information makes exosomes fundamental players in how cells and tissues respond to duress, positioning them as ideal candidates for a “liquid biopsy” that can report on ongoing cellular pathophysiology (Figure 1).

Despite the compelling theoretical framework for exosome involvement in trauma pathophysiology, current understanding of their specific roles in orthopedic complications remains fragmentary. While extensive research has characterized exosome functions in cancer and cardiovascular disease, the mechanistic contribution of extracellular vesicles to ACS, FES, DVT, and fracture healing complications has received limited systematic investigation. This review synthesizes current mechanistic knowledge of exosome-mediated signaling in these major trauma complications, positioning exosomes as active functional mediators rather than passive biomarkers. We propose exosome-centric mechanistic models for each condition, identify critical knowledge gaps, and establish a conceptual framework for future investigations into vesicle-mediated intercellular communication during post-traumatic tissue responses (Table 1).

**Table 1:** Comparative analysis of biomarkers in orthopedic trauma: from traditional to emerging approaches.

Feature	Traditional biomarkers (CK, LDH, D-dimer)	Cytokines (IL-6, TNF-α, CRP)	Cell-free DNA (cfDNA)	Circulating free miRNAs	Microvesicles (MVs)	Exosomes
Origin	Passive release from cell death; systemic byproduct	Secreted by activated immune cells; acute phase response	Released from dying/damaged cells; nuclear/mitochondrial DNA	Released from cells or vesicle degradation	Cell membrane budding; stress response	Active, regulated secretion via endosomal pathway from living cells
Size	Small molecules (<1 kDa)	Small proteins (15–25 kDa)	DNA fragments (150–200 bp)	Small RNA (18–25 nt)	100–1,000 nm	30–160 nm
Information Content	One-dimensional: single molecule concentration	Limited: protein concentration reflects inflammation	Moderate: quantity reflects cell death; fragmentation patterns	Moderate: specific sequences reflect gene regulation	Multi-dimensional: proteins, RNAs, surface markers	Multi-dimensional: proteins, mRNAs, miRNAs, lipids
Specificity	Low: general indicator of tissue damage or coagulation	Moderate: inflammation-specific but not disease-specific	Low-Moderate: elevated in various injuries; mtDNA more specific	Moderate-High: specific sequences can indicate tissue origin	Moderate-High: cargo varies by cell type and state	High: cargo is highly specific to parent cell type and pathological state
Temporal Dynamics	Lagging: peaks hours to days post-injury	Rapid: peaks within hours but transient	Rapid: released immediately upon cell death	Variable: depends on synthesis and release kinetics	Intermediate: released during ongoing cellular stress	Predictive: cargo changes reflect real-time cellular responses
Stability in Circulation	Variable: enzymatic degradation	Low: rapid clearance, short half-life (minutes-hours)	Low: susceptible to nuclease degradation	Moderate: protein-bound or vesicle-associated forms more stable	Moderate: membrane protection but heterogeneous stability	High: lipid bilayer protects cargo from degradation
Functional Activity	None: end products of metabolism	Moderate: can trigger inflammatory cascades	Limited: may trigger inflammation via TLR9	High: can enter cells and regulate gene expression	High: can transfer bioactive molecules and surface receptors	High: transfers functional cargo; can reprogram recipient cells
Cargo Sorting Mechanism	N/A	N/A	Random: reflects nuclear/mitochondrial content	Limited: selective incorporation into vesicles	Non-selective: content reflects cytoplasmic composition	Highly selective: ESCRT-dependent and -independent pathways
Quantification Complexity	Simple: standard clinical assays	Simple: ELISA, immunoassays	Moderate: qPCR, digital PCR	Moderate: qPCR, sequencing	Complex: requires flow cytometry, nanoparticle tracking	Complex: requires isolation, characterization, cargo analysis
Clinical Validation	Extensive: decades of clinical use	Extensive: widely used in clinical practice	Moderate: increasing use in oncology and sepsis	Emerging: limited clinical standardization	Limited: mostly research applications	Emerging: rapidly growing evidence base
Cost	Low: routine clinical tests	Low-Moderate: standard immunoassays	Moderate: molecular assays required	Moderate-High: specialized RNA analysis	High: specialized equipment needed	High: isolation and multi-omics analysis
Therapeutic Potential	None: purely diagnostic	Limited: anti-cytokine therapies exist but not biomarker-based	None: diagnostic only	Moderate: oligonucleotide therapeutics under development	Moderate: potential engineered delivery vehicles	High: natural drug delivery vehicles; can be engineered for targeted therapy
Limitations	Non-specific; late indicators; cannot identify injury source	Transient elevation; influenced by multiple factors; poor specificity	Short half-life; requires rapid processing; background noise	Lack of standardization; multiple sources; stability issues	Heterogeneous population; difficult to distinguish from exosomes	Isolation challenges; standardization needed; cost and expertise barriers
Advantages in Trauma	Widely available; cost-effective; established reference ranges	Reflects acute inflammation; rapid response	Quantifies cell death; differentiates necrosis from apoptosis	Stable in biofluids; tissue-specific signatures	Procoagulant activity measurable; reflects cellular activation	Comprehensive molecular snapshot; predictive value; therapeutic target and vehicle

## 2 Exosome-mediated signaling in acute compartment syndrome: ischemia–reperfusion injury and hypoxic cellular communication

### 2.1 Pathophysiological foundation of acute compartment syndrome

Acute compartment syndrome represents a devastating orthopedic emergency characterized by elevated pressure within a closed fascial compartment, resulting in compromised tissue perfusion and subsequent ischemia–reperfusion injury (10, 11). The pathophysiology involves a self-perpetuating cascade where trauma-induced hemorrhage and edema elevate intracompartmental pressure beyond venous capillary pressure, impeding venous outflow and further exacerbating pressure increases. This progression ultimately compromises arterial inflow, creating a state of profound tissue hypoxia within the affected compartment (12, 13). The temporal progression of ACS follows a biphasic pattern of injury: initial ischemic damage followed by reperfusion injury upon surgical decompression, which paradoxically exacerbates cellular dysfunction through reactive oxygen species generation and sterile inflammation (14, 15).

Current diagnostic approaches rely predominantly on clinical examination, particularly the hallmark sign of pain disproportionate to injury, supplemented by invasive compartment pressure measurements (16, 17). However, these clinical manifestations often emerge only after significant tissue damage has occurred, highlighting the critical need for molecular indicators capable of detecting early ischemic changes before irreversible cellular injury ensues. The cellular environment during ACS progression involves complex intercellular communication networks that orchestrate tissue responses to hypoxia, positioning extracellular vesicles as potential mediators and reporters of this pathological process.

### 2.2 Hypoxia-inducible factor-1α and exosomal reprogramming

Tissue hypoxia serves as the central pathophysiological trigger in ACS and represents a potent stimulus for cellular exosome biogenesis and cargo modification (18, 19). Under hypoxic conditions, hypoxia-inducible factor-1α (HIF-1α) escapes proteasomal degradation, accumulates in the nucleus, and activates transcriptional programs essential for cellular adaptation to oxygen deprivation (20, 21). Beyond its established roles in angiogenesis and metabolic reprogramming, HIF-1α directly regulates genes involved in exosome biogenesis and cargo sorting, fundamentally altering both the quantity and molecular composition of secreted extracellular vesicles (22, 23).

Recent investigations have demonstrated that hypoxic stress significantly enhances exosome secretion rates while profoundly restructuring their proteomic and nucleic acid profiles, particularly enriching them with hypoxia-responsive microRNAs termed “hypoxamiRs” (24, 25). In the context of compartment syndrome, skeletal myocytes and vascular endothelial cells represent the primary cellular sources of these hypoxia-modified exosomes. Ischemic myocytes release exosomes that reflect their metabolic distress and structural damage, while endothelial cells, positioned at the interface of systemic circulation and tissue perfusion, secrete vesicles carrying distinct signatures of vascular dysfunction and inflammatory activation (26, 27). This cellular response creates a systemic molecular fingerprint of localized tissue hypoxia that can potentially be detected in peripheral circulation before clinical symptoms manifest.

### 2.3 Molecular signatures of hypoxic exosomes in ischemia–reperfusion injury

The molecular cargo of exosomes released during ischemia–reperfusion injury undergoes systematic remodeling that reflects the underlying pathophysiological processes. Hypoxia-responsive microRNAs constitute a particularly informative component of this cargo, with specific hypoxamiRs serving as sensitive indicators of cellular oxygen deprivation. HIF-1α-regulated miR-210 represents a well-characterized hypoxamir that promotes cellular survival during oxygen limitation and is consistently packaged into exosomes from hypoxic cells across multiple tissue types (28, 29). Similarly, endothelial-specific miR-424 facilitates angiogenic responses by stabilizing HIF-*α* subunits, creating positive feedback loops that amplify hypoxic signaling (30). Muscle-specific microRNAs, including miR-1 and miR-206, undergo dysregulation during skeletal muscle ischemia and are subsequently enriched in circulating exosomes, providing tissue-specific indicators of myocyte distress (31, 32).

Proteomic analysis of ischemia–reperfusion-derived exosomes reveals systematic alterations in protein cargo that mirror the underlying metabolic and inflammatory changes. Studies utilizing experimental models of limb ischemia have identified enrichment of metabolic enzymes, structural proteins, and inflammatory mediators in post-ischemic exosomes, reflecting the cellular stress response and tissue degradation processes (33, 34). Recent work using advanced proteomics approaches has demonstrated that exosomes from hypoxic endothelial cells carry distinct protein signatures related to coagulation activation, inflammatory signaling, and vascular repair mechanisms (35, 36). These findings establish that ischemia–reperfusion injury induces systematic changes in exosomal molecular composition that can serve as proximal indicators of tissue-level pathophysiology, offering advantages over traditional damage markers that reflect terminal cellular injury events.

### 2.4 A mechanistic model: exosomes as propagators of injury in ACS

Based on the evidence, we propose a mechanistic model wherein exosomes are not merely passive reporters but active participants in the progression of ACS. In the initial ischemic phase, distressed myocytes and endothelial cells release a primary wave of exosomes carrying a HIF-1α-driven hypoxic signature. These exosomes function as nanoscale danger-associated molecular patterns (DAMPs) upon entering the local tissue microenvironment and systemic circulation. They can signal to adjacent, less-affected cells, propagating the injury signal and priming them for subsequent damage. Furthermore, they can recruit and activate inflammatory leukocytes, such as neutrophils and macrophages, amplifying the sterile inflammatory response that is a hallmark of ischemia–reperfusion injury (IRI). This exosome-mediated propagation of danger signals creates a feed-forward loop that accelerates the progression toward irreversible tissue damage. This model reframes exosomes as central players in the ACS cascade, offering a unique window to monitor the otherwise invisible, early-stage molecular pathology and providing a compelling rationale for their development as next-generation diagnostic tools.

## 3 Adipocyte-derived exosomes: mediators of endothelial dysfunction in fat embolism syndrome

### 3.1 FES as a pathology of ectopic adipose signaling

Fat Embolism Syndrome (FES) is a rare but life-threatening complication predominantly associated with long-bone fractures, characterized by a triad of respiratory distress, neurological impairment, and petechial rash (37, 38). Historically, its pathophysiology was primarily attributed to the “mechanical theory,” which posits that large fat globules liberated from the disrupted bone marrow physically obstruct the pulmonary and systemic microvasculature (39). While mechanical occlusion undoubtedly contributes to the initial insult, this theory alone fails to adequately explain the delayed onset of symptoms and the profound systemic inflammatory response that defines the clinical syndrome. A more comprehensive understanding is offered by the “biochemical theory,” which reframes FES as a systemic pathology driven by the toxic effects of fat-derived mediators (37, 40). Following trauma, intramedullary fat enters the venous circulation, where it is hydrolyzed by lipoprotein lipase into free fatty acids (FFAs). These FFAs exert direct cytotoxic effects on the pulmonary endothelium, disrupting alveolar-capillary membrane integrity and promoting the development of non-cardiogenic pulmonary edema, a condition clinically similar to Acute Respiratory Distress Syndrome (ARDS) (37, 41). This biochemical injury cascade, which triggers a systemic inflammatory response syndrome (SIRS), is now considered the central driver of FES pathology. This perspective shifts the focus from simple mechanical blockade to a complex process of ectopic adipose signaling, where mediators released from fat tissue orchestrate a systemic disease process.

### 3.2 Adipocyte-to-endothelial communication via exosomes

In this context of ectopic adipose signaling, adipocyte-derived exosomes (ADEs) are emerging as critical mediators. Adipose tissue is no longer viewed as a passive energy reservoir but as a highly active endocrine organ that communicates with distant tissues via secreted factors, including exosomes (42, 43). Following a major fracture, the massive disruption of bone marrow adipocytes releases not only lipid droplets but also a profound bolus of ADEs into the circulation. These vesicles serve as sophisticated carriers of bioactive cargo, capable of modulating the function of recipient cells, with the vascular endothelium being a primary target (44).

A growing body of evidence, largely from studies on metabolic diseases such as obesity and type 2 diabetes, demonstrates that ADEs from pathological adipose tissue can potently induce endothelial dysfunction. For instance, experimental studies have shown that extracellular vesicles from inflamed and hypoxic adipocytes significantly impair endothelial cell function, with inflammatory ADEs causing up to 4.2-fold increases in vascular cell adhesion molecule-1 (VCAM-1) expression compared to basal levels (45). Mechanistically, these ADEs can transfer specific microRNAs that suppress key endothelial protective pathways, such as the nitric oxide (NO) signaling cascade, thereby compromising vasodilation (46). Recent investigations have demonstrated that exosomes derived from diabetic brown adipose tissue significantly downregulate the calcium/calmodulin-dependent protein kinase II (CaMKII)/endothelial nitric oxide synthase (eNOS) pathway, resulting in diminished NO production and impaired endothelium-dependent relaxation (47). Thus, the communication pathway from adipocyte to endothelium via exosomes represents a crucial, yet previously overlooked, axis in the pathogenesis of trauma-induced endotheliopathy.

### 3.3 The pro-inflammatory and bioactive cargo of ADEs

The capacity of ADEs to induce endothelial dysfunction is encoded within their specific molecular cargo, which is actively sorted and packaged based on the state of the parent adipocyte. This cargo includes a diverse array of proteins, lipids, and nucleic acids that can collectively trigger an inflammatory and injurious response in endothelial cells. Among the most studied components are microRNAs, particularly miR-155, a well-characterized pro-inflammatory miRNA that has been found to be enriched in ADEs from obese adipose tissue macrophages. Upon delivery to endothelial cells or macrophages, exosomal miR-155 can promote a pro-inflammatory phenotype and contribute to insulin resistance through modulation of peroxisome proliferator-activated receptor gamma (PPARγ) pathways, exacerbating vascular inflammation (48–50).

Beyond miRNAs, ADEs can also transport pro-inflammatory cytokines such as tumor necrosis factor-alpha (TNF-*α*) and interleukin-6 (IL-6), which can directly activate inflammatory signaling pathways like nuclear factor-κB (NF-κB) in recipient endothelial cells (51, 52). Furthermore, ADEs facilitate the upregulation of intercellular adhesion molecule-1 (ICAM-1) and vascular cell adhesion molecule-1 (VCAM-1) on endothelial surfaces. This heightened expression of adhesion molecules facilitates the recruitment and transmigration of leukocytes, a critical step in initiating and amplifying the local and systemic inflammation characteristic of FES (53, 54). The lipid composition of the exosomal membrane itself, particularly the exposure of certain bioactive lipids and the coordinated action of inflammatory mediators including inducible nitric oxide synthase (iNOS), can also contribute to the inflammatory cascade (55). This complex payload ensures that ADEs released from a trauma site act as potent, multi-pronged signaling packages that deliver a concentrated pro-inflammatory and pro-thrombotic signal directly to the vascular endothelium.

### 3.4 A mechanistic model: adipocyte exosomes as the “first hit” in FES pathogenesis

Integrating these findings, we propose a refined, exosome-centric mechanistic model for the pathogenesis of FES. In this model, ADEs act as the “first hit” in a multi-step injury process. Immediately following fracture, the massive release of ADEs from the bone marrow precedes the bulk hydrolysis of fat globules into FFAs. These ADEs travel rapidly through the circulation to the lungs, where they encounter and are taken up by the pulmonary capillary endothelium. The delivery of their pro-inflammatory cargo primes the endothelium, shifting it to an activated, dysfunctional state characterized by increased permeability, heightened expression of adhesion molecules, and a pro-coagulant surface. This “first hit” renders the pulmonary microvasculature hyper-susceptible to subsequent insults. The “second hit” arrives as the large neutral fat emboli are progressively hydrolyzed, leading to a surge in circulating FFAs. These toxic FFAs inflict direct chemical injury upon the already-primed and vulnerable endothelium. This two-hit sequence, initiated by ADEs and exacerbated by FFAs, culminates in a catastrophic failure of the alveolar-capillary barrier, massive leukocyte infiltration, and the full clinical manifestation of FES. This model positions ADEs not merely as markers, but as critical early instigators of the disease cascade, offering a novel conceptual framework and identifying a new set of potential targets for therapeutic intervention.

## 4 Exosomes in trauma-induced hypercoagulability and deep vein thrombosis

### 4.1 The cellular basis of trauma-induced coagulopathy

Deep vein thrombosis (DVT) and its potentially fatal sequela, pulmonary embolism (PE), remain among the most feared complications following major orthopedic trauma. The elevated risk in this patient population represents a classic clinical manifestation of Virchow’s triad: venous stasis, endothelial injury, and a systemic hypercoagulable state (56). Trauma-induced coagulopathy (TIC) describes a complex biological response that evolves through distinct temporal phases, with early hypocoagulability typically transitioning to late hypercoagulability within 24 h post-injury (57). This late hypercoagulable state is characterized by a systemic prothrombotic potential that dramatically increases the risk of venous thromboembolism and multiple organ failure (58).

The pathophysiology of TIC originates from severe tissue damage combined with hemorrhagic shock, resulting in widespread endothelial cell activation and dysfunction (59). Extensive tissue injury causes loss of the endothelial glycocalyx and increased tissue factor (TF) expression, which collectively activate coagulation cascades (60). Simultaneously, dying cells release damage-associated molecular patterns (DAMPs), including cell-free DNA, histones, and high mobility group box protein 1 (HMGB-1), that signal tissue injury and trigger systemic inflammatory responses intrinsically linked to coagulation activation (61). This process, termed thromboinflammation, establishes the cellular foundation for trauma-induced hypercoagulability through the coordinated activation of platelets, endothelial cells, and immune system components.

### 4.2 Prothrombotic surfaces on the nanoscale

Following traumatic stress, activated platelets and endothelial cells release substantial quantities of extracellular vesicles into the circulation, creating mobile prothrombotic platforms that dramatically amplify coagulation potential (62). Platelet-derived extracellular vesicles (PEVs) represent the most abundant circulating vesicles in human blood and exhibit significantly enhanced prothrombotic activity compared to their parent cells (63). Recent investigations demonstrate that trauma results in a massive systemic surge of PEVs that contribute to both enhanced hemostasis and pathological thrombosis (62).

The prothrombotic potency of these vesicles derives from two critical molecular features. First, their outer membrane leaflet becomes enriched with phosphatidylserine (PS), providing an ideal anionic surface for assembly of tenase and prothrombinase coagulation complexes (64). Experimental evidence demonstrates that PS exposure on platelet-derived vesicles serves as the primary mechanism for thrombin generation, with this activity efficiently blocked by annexin V but not by anti-tissue factor antibodies (65). Second, extracellular vesicles can carry tissue factor (TF), the primary physiological initiator of the extrinsic coagulation cascade (66). While platelets themselves do not constitutively express TF, they can acquire it from TF-bearing vesicles through fusion mechanisms, creating circulating platforms capable of initiating coagulation throughout the vasculature (67).

### 4.3 Exosome-mediated crosstalk in thrombogenesis

The role of extracellular vesicles extends beyond providing catalytic surfaces to include active participation in complex intercellular communication networks that sustain thromboinflammation. Platelet-derived exosomes can transfer specific cargo, including microRNAs and inflammatory proteins, to endothelial cells, inducing procoagulant phenotypes and promoting expression of adhesion molecules such as ICAM-1 (68). This enables activated platelets to remotely “instruct” distant, uninjured endothelium to become prothrombotic, amplifying the initial local response into a systemic phenomenon.

A particularly compelling example of this crosstalk involves the formation of neutrophil extracellular traps (NETs), web-like structures composed of DNA filaments coated with histones and granule proteins that provide scaffolds for thrombus formation (69). Recent evidence demonstrates that extracellular vesicles can promote NET formation (NETosis), while NET components, particularly histones H3 and H4, can further activate platelets and endothelial cells, creating self-perpetuating feedback loops (70, 71). Studies in trauma patients reveal that NETs are abundant within both arterial and venous thrombi, with NET components serving as both structural scaffolds and activators of the coagulation cascade (72).

The interaction between extracellular vesicles and NETs creates a particularly potent prothrombotic environment. NETs provide physical scaffolds that capture platelets, red blood cells, and coagulation factors, while simultaneously presenting concentrated amounts of histones and DNA that can activate circulating platelets and promote further vesicle release (73). This intricate, vesicle-mediated intercellular dialogue ensures that the initial local response to trauma amplifies into a systemic and self-sustaining prothrombotic state.

### 4.4 A mechanistic model: an exosome-driven “systemic prothrombotic potential”

Based on current evidence, we propose an extracellular vesicle-centric mechanistic model for trauma-induced DVT. Major orthopedic trauma triggers a massive systemic surge of highly procoagulant extracellular vesicles, primarily from activated platelets and endothelial cells, establishing a state of “systemic prothrombotic potential” (74). This state is characterized by elevated circulating concentrations of PS- and TF-bearing vesicles that dramatically lower the threshold for coagulation activation throughout the vasculature.

While this prothrombotic potential does not cause spontaneous thrombosis in high-flow arterial systems, it renders the venous circulation, particularly deep veins with sluggish flow, exquisitely vulnerable to thrombus formation. In areas of venous stasis, circulating procoagulant vesicles have sufficient residence time to initiate and amplify thrombin generation, leading to fibrin deposition and clot propagation (75). This model elegantly explains how localized orthopedic injury can predispose to distant thrombotic events.

Experimental evidence supporting this model comes from studies demonstrating that trauma-derived extracellular vesicles exhibit unique proteomic signatures. Analysis of vesicles from trauma patients who developed DVT revealed profound enrichment with proteins indicative of neutrophil-driven thromboinflammation, including myeloperoxidase (MPO) and histone H4, with levels exceeding 150-fold and 100-fold higher than controls, respectively (76). These proteins represent key components of NETs and serve as direct indicators of the pathogenic cellular processes driving thrombus formation. Rather than measuring downstream products of fibrinolysis like D-dimer, detection of these vesicle-associated proteins offers the potential for mechanism-based diagnostic approaches that directly quantify the upstream drivers of thrombogenesis.

## 5 Exosome-mediated regulation of fracture healing and the pathogenesis of non-union

### 5.1 Fracture repair as a coordinated intercellular dialogue

Fracture healing represents a complex, orchestrated biological process that recapitulates elements of embryonic bone development to restore skeletal integrity (76). This regenerative cascade proceeds through distinct yet overlapping phases, beginning with an inflammatory response, followed by soft callus formation, hard callus formation, and finally bone remodeling (77). The success of this process depends on meticulously coordinated intercellular communication between diverse cell populations, including mesenchymal stem cells (MSCs), osteoblasts, osteoclasts, and endothelial cells (78). This communication network ensures timely recruitment and differentiation of progenitor cells, formation of a vascularized callus, and the delicate balance between bone formation and bone resorption.

Recent advances have revealed that extracellular vesicles, particularly exosomes, serve as critical mediators in this intercellular dialogue, acting as nanoscale messengers that transfer functional cargo to coordinate key events of bone repair (79). When this intricate communication network fails, the healing process stalls, leading to delayed union or atrophic non-union, complications that affect 5–10% of the six million annual fractures in the United States (80). Non-union can thus be fundamentally viewed as a breakdown in the crucial intercellular signaling that governs the entire regenerative process (81).

### 5.2 Exosomal control of osteogenesis and angiogenesis

#### 5.2.1 Pro-osteogenic exosomal signals

The recruitment and differentiation of MSCs into bone-forming osteoblasts represents the cornerstone of fracture repair, and bone marrow mesenchymal stem cell-derived exosomes (BMSC-Exos) have emerged as powerful mediators of this process (82). Experimental evidence demonstrates that BMSC-Exos significantly enhance fracture healing through promotion of both osteogenesis and angiogenesis in animal models of non-union (82). These vesicles carry pro-osteogenic cargo that activates critical signaling pathways in recipient cells through well-characterized molecular mechanisms.

BMSC-Exos promote osteogenesis primarily through activation of the bone morphogenetic protein-2 (BMP-2)/Smad1/runt-related transcription factor 2 (RUNX2) signaling pathway (82). RUNX2 serves as the master transcription factor for osteoblast differentiation, and its expression and activity are tightly regulated by multiple mechanisms, including post-translational modifications and microRNA-mediated control (83). Recent studies have identified specific exosomal microRNAs that regulate RUNX2 function. For example, exosomal miR-25 from BMSCs promotes fracture healing by inhibiting SMURF1-mediated ubiquitination and degradation of RUNX2, thereby enhancing osteoblast differentiation, proliferation, and migration (84).

Conversely, inhibitory microRNAs can impair fracture healing through targeting of osteogenic pathways. Clinical studies have identified that miR-628-3p is upregulated in patients with atrophic non-union and directly targets RUNX2, leading to suppressed osteoblast differentiation (85). Similarly, miR-133a has been shown to inhibit fracture healing through targeting of the RUNX2/BMP2 signaling pathway, with higher expression levels observed in non-union patients compared to those with normal healing (86). These findings establish exosomal microRNAs as critical regulators of the osteogenic response during fracture repair.

#### 5.2.2 Angiogenic coupling

Successful fracture healing requires tight coupling between angiogenesis and osteogenesis, as the formation of new blood vessels is essential for supplying oxygen and nutrients to the developing callus (87). Hypoxic preconditioning of MSCs enhances the pro-angiogenic capacity of their derived exosomes, with hypoxia-conditioned exosomes (Hypo-Exos) demonstrating superior effects on bone fracture healing compared to normoxic exosomes (88). This enhanced functionality is mediated through enrichment of specific microRNAs, particularly miR-126, which promotes endothelial cell proliferation, migration, and angiogenesis through suppression of SPRED1 and activation of the Ras/ERK pathway (88).

BMSC-derived exosomes also promote angiogenesis through the hypoxia-inducible factor-1α (HIF-1α)/vascular endothelial growth factor (VEGF) signaling pathway (82). Additionally, exosomal miR-29a has been identified as a key mediator of the angiogenic-osteogenic coupling process. BMSC-derived exosomal miR-29a promotes both angiogenesis and osteogenesis by targeting VASH1 (vasohibin 1), leading to enhanced endothelial cell proliferation, migration, and tube formation, as well as increased osteoblast function (89). This dual functionality demonstrates how single exosomal components can coordinate multiple aspects of the fracture healing process.

### 5.3 Exosomal control of osteoclastogenesis and bone remodeling

The final phase of fracture healing involves remodeling of the hard callus into mature lamellar bone, a process requiring precisely controlled balance between bone formation by osteoblasts and bone resorption by osteoclasts (90). This balance is regulated through the well-characterized RANKL (receptor activator of nuclear factor κB ligand)/RANK/osteoprotegerin (OPG) signaling axis, where osteoblasts secrete RANKL to stimulate osteoclast differentiation and activation (91).

Recent evidence indicates that this classical communication pathway is supplemented by extracellular vesicle-mediated signaling. Osteoblast-derived vesicles can carry RANKL and deliver it directly to osteoclast precursors, while osteoclasts can release vesicles containing RANK that may activate reverse signaling pathways in osteoblasts (92). Furthermore, osteoclast-derived extracellular vesicles contain specific microRNAs that can influence osteoblast behavior. For instance, miR-324 present in osteoclast-derived vesicles promotes osteogenic differentiation through downregulation of ARHGAP1 and activation of RhoA/ROCK signaling (93).

During fracture healing, endogenous parathyroid hormone (PTH) signaling demonstrates the complex regulation of osteoclast–osteoblast communication. PTH deficiency affects osteoclast activity by reducing RANKL expression in osteoblasts through the PI3K/AKT/STAT5 pathway (94). This illustrates how systemic hormonal signals can modulate local exosomal communication networks to influence fracture healing outcomes.

### 5.4 A mechanistic model for non-union: the “inhibitory exosomal signature”

Based on accumulating evidence, we propose a mechanistic model for atrophic non-union centered on dysregulated exosomal communication networks. In normal fracture healing, the circulating and local exosome populations undergo dynamic temporal changes that reflect and drive the healing process. Following the initial inflammatory phase, successful healing requires establishment of a “pro-regenerative exosomal signature” characterized by high levels of exosomes carrying pro-osteogenic and pro-angiogenic cargo from MSCs, osteoblasts, and other supportive cell types (95).

Atrophic non-union may arise from failure to establish and maintain this pro-regenerative exosomal environment. Clinical evidence supporting this hypothesis comes from studies demonstrating that plasma exosomes derived from infected fracture non-union patients delay fracture repair in experimental models by inhibiting osteogenic differentiation of bone marrow stromal cells (96). These pathological exosomes exhibit distinct microRNA profiles, including overexpression of miR-708-5p, which targets structure-specific recognition protein 1 (SSRP1) and suppresses the Wnt/*β*-catenin signaling pathway, thereby impairing osteogenic differentiation (96).

This model suggests that the molecular signature of circulating exosomes could serve as a dynamic biomarker for monitoring fracture healing progression. Early detection of inhibitory exosomal signatures—such as persistent elevation of anti-osteogenic microRNAs or failure to detect rising levels of pro-regenerative factors—could provide mechanism-based early warning signs for impending non-union, enabling intervention before radiological evidence of healing failure becomes apparent.

**Figure 2:**
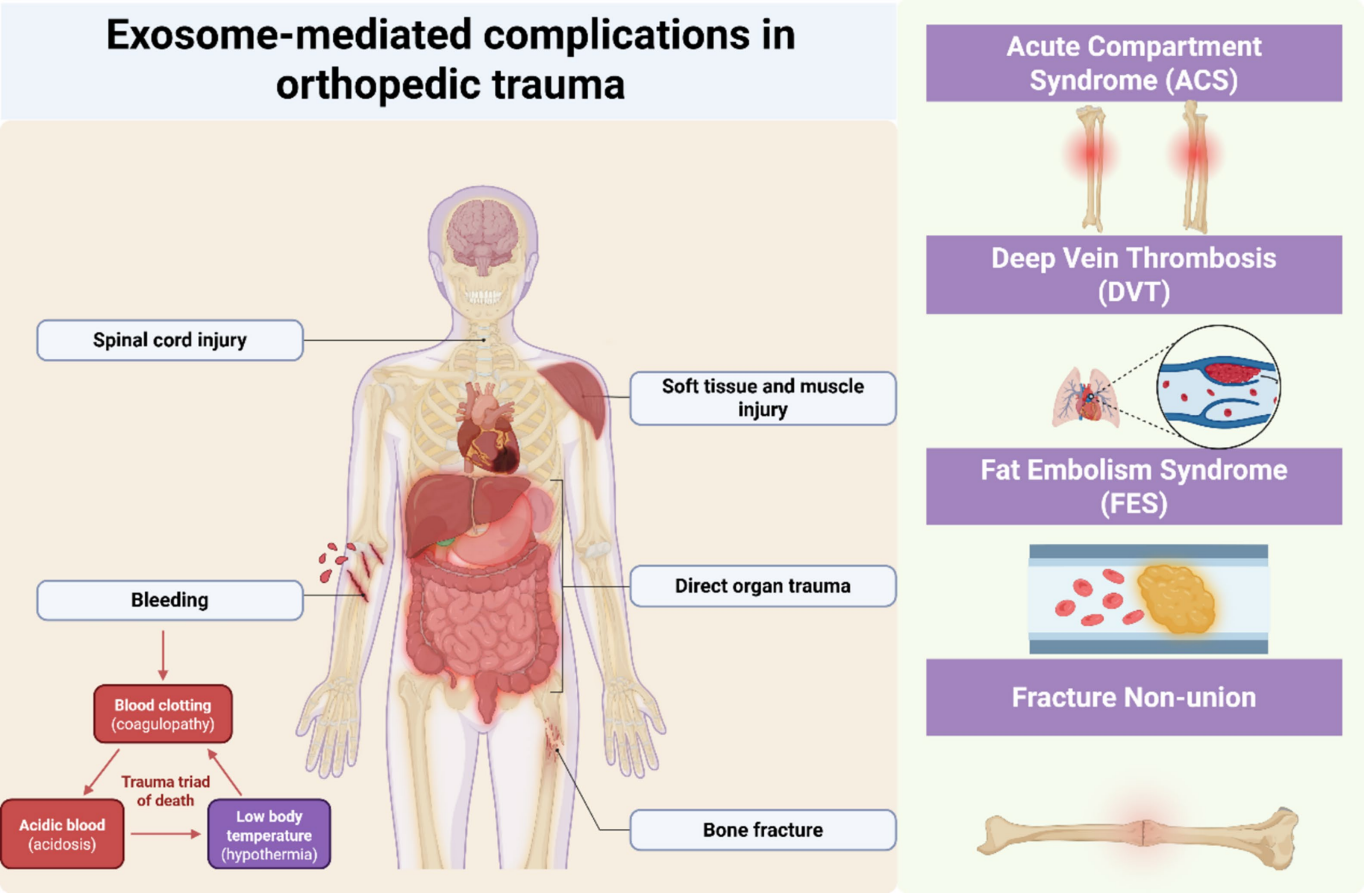
Exosome-mediated complications in orthopedic trauma. Schematic showing exosome-mediated mechanisms in four orthopedic trauma complications: ACS (ischemia–reperfusion, HIF-1α pathway), FES (adipocyte exosomes, endothelial dysfunction), DVT (hypercoagulable state, platelet activation), and fracture non-union (bone repair dysregulation, osteogenic signaling). Exosomes function as active mediators rather than passive biomarkers in trauma-related pathophysiology.

The therapeutic implications of this model extend beyond biomarker development to direct intervention strategies. Exosome-based therapies, including BMSC-derived exosomes loaded with specific pro-osteogenic microRNAs or growth factors, have demonstrated efficacy in preclinical fracture healing models and represent promising cell-free therapeutic approaches that avoid the complications associated with direct stem cell transplantation (97–99) (Table 2; Figure 2).

**Table 2:** Key exosomal biomarker candidates and their roles in orthopedic trauma complications.

Complication	Cellular origin	Biomarker candidate	Molecular mechanism	Clinical implication	Study type	Animal model	Key references
Acute compartment syndrome (ACS)	Skeletal Myocytes, Endothelial Cells	miR-210	Key mediator of cellular adaptation to hypoxia.	Direct indicator of severe tissue hypoxia.	*In vitro* (renal cells)	Not specified	(28, 29)
		miR-424	Promotes angiogenesis by stabilizing HIF-α.	Marker of endothelial response to ischemia.	*In vitro* (human endothelial cells)	Not specified	(30)
	Skeletal Myocytes	miR-1, miR-206 (MyomiRs)	Myo-specific miRNAs released upon cellular distress.	Tissue-specific marker of muscle damage.	Clinical (serum from patients)	Not applicable	(31, 32)
Fat embolism syndrome (FES)	Adipocytes, Macrophages	miR-155	Promotes a pro-inflammatory M1 macrophage phenotype.	Indicator of systemic inflammatory potential.	*In vitro* and *in vivo*	Mice	(48–50)
		TNF-α, IL-6	Pro-inflammatory cytokines that activate NF-κB pathways.	Direct measure of the inflammatory payload.	Review/mechanistic	Not applicable	(51, 52)
Deep vein thrombosis (DVT)	Platelets, Endothelial Cells	PS & TF (Surface)	Provides catalytic surface & initiates coagulation cascade.	Direct measure of systemic procoagulant potential.	*In vitro* and *in vivo*	Mice	(64–67)
	Neutrophils	MPO, Histone H4	Key components of Neutrophil Extracellular Traps (NETs).	Specific markers of active thromboinflammation.	Clinical (trauma patients)	Not applicable	(76)
	Neutrophils, Monocytes	S100-A9	DAMP that promotes inflammation and thrombosis.	Indicator of a heightened thromboinflammatory state.	Clinical (trauma patients)	Not applicable	(76)
Fracture non-union	Bone Marrow MSCs	miR-25	Promotes osteogenesis via inhibition of RUNX2 degradation.	Positive predictor of bone healing potential.	*In vivo*	Mice	(84)
	Hypoxic MSCs	miR-126	Promotes angiogenesis via the Ras/ERK pathway.	Marker of a pro-regenerative angiogenic response.	*In vitro* and *in vivo*	Mice	(88)
	BMSCs	miR-29a	Promotes both angiogenesis & osteogenesis by targeting VASH1.	Indicator of angiogenic-osteogenic coupling.	*In vitro* AND *in vivo*	Mice (C57BL/6 J)	(89)
	Inflammatory cells; Pathological tissue	miR-628-3p / miR-133a	Suppresses osteoblast differentiation by targeting RUNX2.	Negative predictors; markers of healing inhibition.	Clinical + *in vitro*	Not applicable (human study)	(85, 86)
	Infected tissue	miR-708-5p	Inhibits osteogenesis by suppressing the Wnt/*β*-catenin pathway.	Specific marker for infected non-union pathology.	*In vivo* (mouse model)	Mice	(96)

DAMP, damage-associated molecular pattern; MSCs, mesenchymal stem cells; SEC, size-exclusion chromatography.

## 6 Fundamental challenges and future research directions

**Figure 3:**
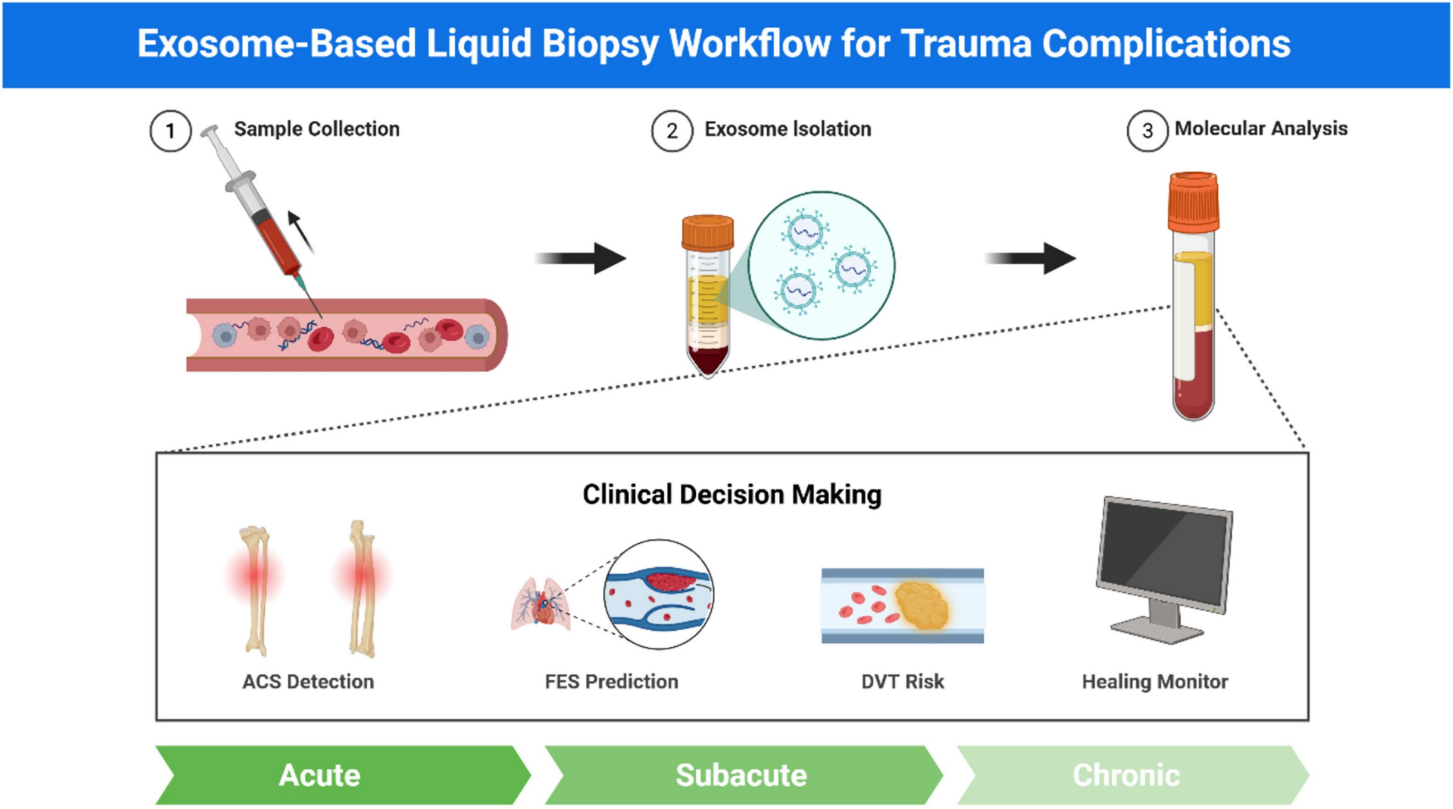
Exosome-based liquid biopsy workflow for trauma complications. Schematic workflow of exosome-based liquid biopsy showing three sequential steps: sample collection from blood/plasma, exosome isolation using standard methods (ultracentrifugation, immunocapture, size exclusion), and molecular analysis including proteomics, miRNA profiling, and surface markers (CD9, CD63, CD81). Clinical applications encompass four trauma complications: ACS detection, FES prediction, DVT risk assessment, and fracture healing monitoring. This approach enables non-invasive, real-time monitoring with functional insights for personalized trauma management across acute, subacute, and chronic phases.

Despite the compelling theoretical framework and emerging evidence supporting exosomes as functional mediators in orthopedic trauma, the field faces critical methodological and conceptual challenges that must be addressed to advance clinical translation. This section examines these challenges systematically and outlines strategic research priorities necessary to unlock the full potential of exosome-based diagnostics and therapeutics (Figure 3).

### 6.1 Methodological standardization and reproducibility

The greatest impediment to exosome research advancement lies in the fundamental lack of methodological standardization across isolation, characterization, and analysis protocols. Current practices encompass diverse approaches including ultracentrifugation, size-exclusion chromatography, polymer-based precipitation, and immunoaffinity capture, each yielding preparations with varying purity, yield, and contamination profiles (100). This methodological heterogeneity severely compromises inter-study reproducibility and prevents meaningful meta-analyses of existing literature.

The International Society for Extracellular Vesicles has addressed this challenge through the recently updated Minimal Information for Studies of Extracellular Vesicles (MISEV2023) guidelines, which represent a consensus from over 1,000 researchers worldwide (101). These guidelines establish rigorous criteria for vesicle characterization, emphasizing the need for multiple orthogonal approaches to confirm vesicle identity and purity. Adherence to MISEV2023 standards is no longer optional but represents a prerequisite for generating scientifically valid and clinically relevant data.

Beyond standardization, the field urgently requires development of high-throughput, clinically feasible isolation methods that can process large patient cohorts without compromising vesicle integrity or introducing systematic biases. Current gold-standard techniques such as differential ultracentrifugation, while effective for research applications, lack the scalability and standardization necessary for clinical implementation (102).

### 6.2 Addressing vesicle heterogeneity through single-particle analysis

Blood plasma contains an extraordinarily complex mixture of extracellular vesicles originating from diverse cellular sources throughout the body. This inherent heterogeneity presents a fundamental challenge for identifying pathologically relevant vesicle populations within the total circulating pool. Traditional bulk analysis approaches provide only averaged signals across entire populations, obscuring critical information about specific subsets that may carry disease-relevant molecular signatures (103).

The solution lies in emerging single-vesicle analysis technologies that enable phenotypic characterization of individual vesicles. Advanced flow cytometric approaches, including imaging flow cytometry and high-sensitivity instruments optimized for submicron particle detection, now permit multiparametric analysis of individual vesicles in complex biological fluids (104, 105). These platforms can simultaneously assess vesicle size, membrane composition, and surface marker expression, enabling identification and quantification of specific vesicle subpopulations.

Complementing flow cytometric approaches, immunoaffinity capture using cell-type-specific surface markers offers targeted enrichment strategies for isolating vesicles from pathologically relevant sources (106). For orthopedic trauma applications, markers specific for myocytes (e.g., myosin), endothelial cells (e.g., CD31), platelets (e.g., CD61), and osteoblasts (e.g., alkaline phosphatase) enable selective analysis of vesicles from tissues most directly involved in trauma pathophysiology.

### 6.3 Integrative multi-omics approaches for comprehensive characterization

The biological significance of exosomes cannot be captured through single-molecule analysis but requires comprehensive characterization of their entire molecular cargo. The field must transition from reductive approaches focusing on individual biomarkers toward integrative multi-omics platforms that simultaneously profile proteomes, transcriptomes, and lipidomes within the same vesicle populations (107, 108).

Recent technological advances have demonstrated the feasibility of such integrative approaches. Combined proteomic and lipidomic analysis of COVID-19 patient exosomes revealed disease stage-specific molecular signatures that reflected both parent cell pathophysiology and vesicle functional capacity (109). Similarly, comprehensive proteomic analysis of mesenchymal stem cell-derived exosomes from different tissue sources identified distinct functional profiles that correlated with their therapeutic efficacy (110).

For trauma applications, multi-omics characterization could reveal the dynamic molecular evolution of exosomal cargo throughout the post-injury timeline, identifying early signatures predictive of complications before clinical manifestation. Advanced bioinformatics approaches, including machine learning algorithms and network analysis, are essential for extracting meaningful biological insights from these high-dimensional datasets (111).

### 6.4 Functional validation and mechanistic proof-of-concept

The majority of current exosome research establishes correlative relationships between vesicle cargo and clinical outcomes without demonstrating direct causal mechanisms. Advancing the field requires rigorous functional validation demonstrating that specific exosome populations can directly induce the pathophysiological changes they are proposed to mediate (112).

This necessitates sophisticated experimental designs that isolate specific vesicle populations from trauma models and test their biological effects in relevant *in vitro* systems. For compartment syndrome research, this might involve applying hypoxic myocyte-derived exosomes to endothelial barrier function assays or ischemia–reperfusion injury models. For thrombosis studies, platelet-derived vesicles from trauma patients could be tested for their capacity to initiate coagulation in controlled systems (113).

The ultimate validation requires *in vivo* proof-of-concept studies demonstrating that exosomes isolated from trauma conditions can reproduce pathological changes when administered to naive animal models. Such studies would establish definitively whether exosomes function as active disease mediators rather than passive biomarkers (114).

### 6.5 Advanced bio-imaging for *in vivo* vesicle tracking

Understanding the physiological roles of trauma-induced exosomes requires direct visualization of their biodistribution, target cell interactions, and cargo transfer in living systems. Recent advances in exosome labeling and molecular imaging technologies have made such studies increasingly feasible (115, 116).

Multiple labeling strategies are now available, including fluorescent membrane dyes (e.g., DiD, PKH67), genetic reporters (e.g., CD63-GFP fusions), and radiotracers for PET/SPECT imaging (117, 118). Each approach offers distinct advantages: fluorescent labeling enables high-resolution visualization of vesicle-cell interactions, while radioisotope labeling permits quantitative whole-body biodistribution studies over extended timeframes.

For trauma research, such studies could address fundamental questions about exosome pathophysiology: Do platelet-derived vesicles preferentially accumulate in venous beds prone to thrombosis? How long do myocyte-derived vesicles persist in circulation following muscle injury? Which organs take up adipocyte-derived vesicles following fat embolism, and how does this correlate with clinical symptom onset? (119, 120).

### 6.6 Translation toward clinical implementation

The ultimate goal of exosome research in orthopedic trauma is clinical translation for improved patient care. This requires addressing practical considerations including assay standardization, regulatory approval pathways, and health economic evaluation. Point-of-care diagnostic platforms capable of rapid exosome analysis in emergency settings represent a critical technological need (121). Furthermore, the field must establish reference standards and quality control materials for exosome-based assays, similar to those used for conventional biomarkers. This includes defining normal ranges, inter-assay variability, and clinical decision thresholds for different trauma complications (122).

## 7 Conclusions and future perspectives

This comprehensive review has established exosomes as critical functional mediators rather than passive biomarkers in the pathophysiology of major orthopedic trauma complications. Through systematic examination of their roles in acute compartment syndrome, fat embolism syndrome, trauma-induced coagulopathy, and fracture non-union, we have demonstrated that exosomes actively participate in intercellular communication networks that determine patient outcomes. The mechanistic models presented herein reframe these nanoscale vesicles as active participants in injury propagation and tissue response coordination, offering unprecedented insights into the molecular basis of post-traumatic complications. In acute compartment syndrome, hypoxia-driven exosomal cargo facilitates ischemia–reperfusion injury through HIF-1α-mediated molecular reprogramming. Fat embolism syndrome emerges as a two-hit process where adipocyte-derived exosomes prime pulmonary endothelium for subsequent free fatty acid injury. Trauma-induced coagulopathy involves massive systemic release of prothrombotic vesicles that establish a state of heightened thrombotic potential throughout the circulation. Fracture non-union reflects dysregulated exosomal communication networks that fail to maintain the pro-regenerative molecular environment necessary for successful bone healing. These mechanistic insights position exosomes as both diagnostic windows into ongoing pathophysiology and potential therapeutic targets for intervention strategies that could fundamentally alter trauma care paradigms.

Future Perspectives in exosome research for orthopedic trauma must address three critical priorities to achieve clinical translation and maximize therapeutic impact. First, the field requires establishment of standardized, high-throughput platforms for exosome isolation and multi-omics characterization that meet stringent clinical requirements for speed, reproducibility, and cost-effectiveness. This technological advancement will enable routine integration of exosome analysis into emergency care protocols, facilitating early detection of complications before clinical manifestation. Second, mechanistic understanding must be deepened through rigorous functional validation studies that definitively establish causal relationships between specific exosome populations and trauma complications, moving beyond correlative observations to demonstrate direct pathophysiological roles. Such studies will validate exosomes as legitimate therapeutic targets and guide development of intervention strategies. Third, advancement of *in vivo* imaging capabilities to visualize exosome behavior in real-time will enable optimization of timing, dosing, and targeting strategies for future therapeutic applications, while providing fundamental insights into vesicle biodistribution and clearance mechanisms. Success in these research priorities will position exosome-based approaches as transformative tools for both understanding and managing the complex pathophysiology of orthopedic trauma, ultimately improving patient outcomes through precision medicine approaches that target the molecular mediators of post-traumatic complications. The integration of these advances promises to revolutionize trauma care by providing clinicians with molecular-level insights that enable proactive, mechanism-based interventions rather than reactive treatments of established complications.

## Glossary

ACS: Acute Compartment Syndrome
ADEs: Adipocyte-Derived Exosomes
ARDS: Acute Respiratory Distress Syndrome
BMP-2: Bone Morphogenetic Protein-2
BMSC-Exos: Bone Marrow Mesenchymal Stem Cell-Derived Exosomes
CaMKII: Calcium/Calmodulin-Dependent Protein Kinase II
CK: Creatine Kinase
DAMPs: Danger-Associated Molecular Patterns
DVT: Deep Vein Thrombosis
eNOS: Endothelial Nitric Oxide Synthase
ESCRT: Endosomal Sorting Complex Required for Transport
FES: Fat Embolism Syndrome
FFAs: Free Fatty Acids
HIF-1α: Hypoxia-Inducible Factor-1α
HMGB-1: High Mobility Group Box Protein 1
Hypo-Exos: Hypoxia-Conditioned Exosomes
ICAM-1: Intercellular Adhesion Molecule-1
IL-6: Interleukin-6
iNOS: Inducible Nitric Oxide Synthase
IRI: Ischemia–Reperfusion Injury
LDH: Lactate Dehydrogenase
MISEV2023: Minimal Information for Studies of Extracellular Vesicles 2023
MPO: Myeloperoxidase
MSCs: Mesenchymal Stem Cells
NETs: Neutrophil Extracellular Traps
NF-κB: Nuclear Factor-κB
NO: Nitric Oxide
OPG: Osteoprotegerin
PE: Pulmonary Embolism
PEVs: Platelet-Derived Extracellular Vesicles
PPARγ: Peroxisome Proliferator-Activated Receptor Gamma
PS: Phosphatidylserine
PTH: Parathyroid Hormone
RANK: Receptor Activator of Nuclear Factor κB
RANKL: Receptor Activator of Nuclear Factor κB Ligand
RUNX2: Runt-Related Transcription Factor 2
SIRS: Systemic Inflammatory Response Syndrome
SSRP1: Structure-Specific Recognition Protein 1
TF: Tissue Factor
TIC: Trauma-Induced Coagulopathy
TNF-*α*: Tumor Necrosis Factor-Alpha
VASH1: Vasohibin 1
VCAM-1: Vascular Cell Adhesion Molecule-1
VEGF: Vascular Endothelial Growth Factor

## References

[1] Lord JM Midwinter MJ Chen YF Belli A Brohi K Kovacs EJ. The systemic immune response to trauma: an overview of pathophysiology and treatment. Lancet. (2014) 384:1455–65. doi: 10.1016/S0140-6736(14)60687-5, PMID: 25390327 PMC4729362

[2] Keel M Trentz O. Pathophysiology of polytrauma. Injury. (2005) 36:691–709. doi: 10.1016/j.injury.2004.12.037, PMID: 15910820

[3] Pape HC Giannoudis P Krettek C. The timing of fracture treatment in polytrauma patients: relevance of damage control orthopedic surgery. Am J Surg. (2002) 183:622–9. doi: 10.1016/S0002-9610(02)00865-6, PMID: 12095590

[4] de Meijer AR Fikkers BG de Keijzer MH van Engelen BG Drenth JP. Serum creatine kinase as predictor of clinical course in rhabdomyolysis: a 5-year intensive care survey. Intensive Care Med. (2003) 29:1121–5. doi: 10.1007/s00134-003-1800-5, PMID: 12768237

[5] Stein PD Hull RD Patel KC Olson RE Ghali WA Brant R. D-dimer for the exclusion of acute venous thrombosis and pulmonary embolism: a systematic review. Ann Intern Med. (2004) 140:589–602. doi: 10.7326/0003-4819-140-8-200404200-00005, PMID: 15096330

[6] Wakai A Gleeson A Winter D. Role of fibrin D-dimer testing in emergency medicine. Emerg Med J. (2003) 20:319–25. doi: 10.1136/emj.20.4.319, PMID: 12835339 PMC1726151

[7] Arya SB Collie SP Parent CA. The ins-and-outs of exosome biogenesis, secretion, and internalization. Trends Cell Biol. (2024) 34:90–108. doi: 10.1016/j.tcb.2023.06.006, PMID: 37507251 PMC10811273

[8] Rashed MH Bayraktar E Helal GK Abd-Ellah MF Amero P Chavez-Reyes A. Exosomes: from garbage bins to promising therapeutic targets. Int J Mol Sci. (2017) 18:538. doi: 10.3390/ijms18030538 28257101 PMC5372554

[9] Kalluri R LeBleu VS. The biology, function, and biomedical applications of exosomes. Science (New York, NY). (2020) 367:eaau6977. doi: 10.1126/science.aau6977, PMID: 32029601 PMC7717626

[10] Donaldson J Haddad B Khan WS. The pathophysiology, diagnosis and current management of acute compartment syndrome. Open Orthop J. (2014) 8:185–93. doi: 10.2174/1874325001408010185, PMID: 25067973 PMC4110398

[11] von Keudell AG Weaver MJ Appleton PT Bae DS Dyer GSM Heng M. Diagnosis and treatment of acute extremity compartment syndrome. Lancet. (2015) 386:1299–310. doi: 10.1016/S0140-6736(15)00277-9, PMID: 26460664

[12] Schmidt AH. Acute compartment syndrome. Injury. (2017) 48:S22–s25. doi: 10.1016/j.injury.2017.04.024, PMID: 28449851

[13] Garner MR Taylor SA Gausden E Lyden JP. Compartment syndrome: diagnosis, management, and unique concerns in the twenty-first century. HSS J. (2014) 10:143–52. doi: 10.1007/s11420-014-9386-8, PMID: 25050098 PMC4071472

[14] Eltzschig HK Eckle T. Ischemia and reperfusion--from mechanism to translation. Nat Med. (2011) 17:1391–401. doi: 10.1038/nm.2507, PMID: 22064429 PMC3886192

[15] Kalogeris T Baines CP Krenz M Korthuis RJ. Ischemia/reperfusion. Compr Physiol. (2016) 7:113–70. doi: 10.1002/cphy.c160006, PMID: 28135002 PMC5648017

[16] Ulmer T. The clinical diagnosis of compartment syndrome of the lower leg: are clinical findings predictive of the disorder? J Orthop Trauma. (2002) 16:572–7. doi: 10.1097/00005131-200209000-00006, PMID: 12352566

[17] Whitesides TE Heckman MM. Acute compartment syndrome: update on diagnosis and treatment. J Am Acad Orthop Surg. (1996) 4:209–18. doi: 10.5435/00124635-199607000-00005, PMID: 10795056

[18] Mathieu M Martin-Jaular L Lavieu G Théry C. Specificities of secretion and uptake of exosomes and other extracellular vesicles for cell-to-cell communication. Nat Cell Biol. (2019) 21:9–17. doi: 10.1038/s41556-018-0250-9, PMID: 30602770

[19] Hessvik NP Llorente A. Current knowledge on exosome biogenesis and release. Cell Mol Life Sci. (2018) 75:193–208. doi: 10.1007/s00018-017-2595-9, PMID: 28733901 PMC5756260

[20] Semenza GL. Regulation of oxygen homeostasis by hypoxia-inducible factor 1. Physiology (Bethesda). (2009) 24:97–106. doi: 10.1152/physiol.00045.2008, PMID: 19364912

[21] Lee P Chandel NS Simon MC. Cellular adaptation to hypoxia through hypoxia inducible factors and beyond. Nat Rev Mol Cell Biol. (2020) 21:268–83. doi: 10.1038/s41580-020-0227-y, PMID: 32144406 PMC7222024

[22] Bijnsdorp IV van Royen ME Verhaegh GW Martens-Uzunova ES. The non-coding transcriptome of prostate Cancer: implications for clinical practice. Mol Diagn Ther. (2017) 21:385–400. doi: 10.1007/s40291-017-0271-2, PMID: 28299719 PMC5511609

[23] Panigrahi GK Praharaj PP Kittaka H Mridha AR Black OM Singh R. Exosome proteomic analyses identify inflammatory phenotype and novel biomarkers in African American prostate cancer patients. Cancer Med. (2019) 8:1110–23. doi: 10.1002/cam4.1885, PMID: 30623593 PMC6434210

[24] Wang N Chen C Yang D Liao Q Luo H Wang X. Mesenchymal stem cells-derived extracellular vesicles, via miR-210, improve infarcted cardiac function by promotion of angiogenesis. Biochim Biophys Acta Mol basis Dis. (2017) 1863:2085–92. doi: 10.1016/j.bbadis.2017.02.023, PMID: 28249798

[25] Fernández-Messina L Rodríguez-Galán A de Yébenes VG Gutiérrez-Vázquez C Tenreiro S Seabra MC. Transfer of extracellular vesicle-microRNA controls germinal center reaction and antibody production. EMBO Rep. (2020) 21:e48925. doi: 10.15252/embr.201948925, PMID: 32073750 PMC7132182

[26] Guescini M Canonico B Lucertini F Maggio S Annibalini G Barbieri E. Muscle releases alpha-Sarcoglycan positive extracellular vesicles carrying miRNAs in the bloodstream. PLoS One. (2015) 10:e0125094. doi: 10.1371/journal.pone.0125094, PMID: 25955720 PMC4425492

[27] Vechetti IJ Jr Wen Y Chaillou T Murach KA Alimov AP Figueiredo VC. Life-long reduction in myomiR expression does not adversely affect skeletal muscle morphology. Sci Rep. (2019) 9:5483. doi: 10.1038/s41598-019-41476-8, PMID: 30940834 PMC6445125

[28] Liu LL Li D He YL Zhou YZ Gong SH Wu LY. miR-210 protects renal cell against hypoxia-induced apoptosis by targeting HIF-1 alpha. Mol Med. (2017) 23:258–71. doi: 10.2119/molmed.2017.00013, PMID: 29387863 PMC5653737

[29] Devlin C Greco S Martelli F Ivan M. miR-210: more than a silent player in hypoxia. IUBMB Life. (2011) 63:94–100. doi: 10.1002/iub.427, PMID: 21360638 PMC4497508

[30] Ghosh G Subramanian IV Adhikari N Zhang X Joshi HP Basi D. Hypoxia-induced microRNA-424 expression in human endothelial cells regulates HIF-α isoforms and promotes angiogenesis. J Clin Invest. (2010) 120:4141–54. doi: 10.1172/JCI42980, PMID: 20972335 PMC2964978

[31] Koutsoulidou A Kyriakides TC Papadimas GK Christou Y Kararizou E Papanicolaou EZ. Elevated muscle-specific miRNAs in serum of myotonic dystrophy patients relate to muscle disease Progress. PLoS One. (2015) 10:e0125341. doi: 10.1371/journal.pone.0125341, PMID: 25915631 PMC4411125

[32] Sorrentino S Iaconetti C De Rosa S Polimeni A Sabatino J Gareri C. Hindlimb ischemia impairs endothelial recovery and increases Neointimal proliferation in the carotid artery. Sci Rep. (2018) 8:761. doi: 10.1038/s41598-017-19136-6, PMID: 29335599 PMC5768880

[33] Senda A Kojima M Watanabe A Kobayashi T Morishita K Aiboshi J. Profiles of lipid, protein and microRNA expression in exosomes derived from intestinal epithelial cells after ischemia-reperfusion injury in a cellular hypoxia model. PLoS One. (2023) 18:e0283702. doi: 10.1371/journal.pone.0283702, PMID: 36989330 PMC10058167

[34] Zhang Y Liu Y Liu H Tang WH. Exosomes: biogenesis, biologic function and clinical potential. Cell Biosci. (2019) 9:19. doi: 10.1186/s13578-019-0282-2, PMID: 30815248 PMC6377728

[35] Park KS Bandeira E Shelke GV Lässer C Lötvall J. Enhancement of therapeutic potential of mesenchymal stem cell-derived extracellular vesicles. Stem Cell Res Ther. (2019) 10:288. doi: 10.1186/s13287-019-1398-3, PMID: 31547882 PMC6757418

[36] Ginckels P Holvoet P. Oxidative stress and inflammation in cardiovascular diseases and cancer: role of non-coding RNAs. Yale J Biol Med. (2022) 95:129–52. doi: 10.1007/s11883-017-0678-6, PMID: 35370493 PMC8961704

[37] Kawakami D Yoshino S Kawakami S Yamakawa R. Fat embolism syndrome. Intensive Care Med. (2022) 48:748–9. doi: 10.1007/s00134-022-06664-7, PMID: 35286407

[38] Morena D Scopetti M Padovano M Turillazzi E Fineschi V. Fat embolism: a systematic review to facilitate the development of standardised procedures in pathology. Histopathology. (2025) 86:845–61. doi: 10.1111/his.15355, PMID: 39478415 PMC11964584

[39] Rothberg DL Makarewich CA. Fat embolism and fat embolism syndrome. J Am Acad Orthop Surg. (2019) 27:e346–55. doi: 10.5435/JAAOS-D-17-00571, PMID: 30958807

[40] Li Y Shi G Liang W Shang H Li H Han Y. Allogeneic adipose-derived mesenchymal stem cell transplantation alleviates atherosclerotic plaque by inhibiting ox-LDL uptake, inflammatory reaction and endothelial damage in rabbits. Cells. (2023) 12:1936. doi: 10.3390/cells12151936, PMID: 37566014 PMC10417209

[41] Gossling HR Pellegrini VD Jr. Fat embolism syndrome: a review of the pathophysiology and physiological basis of treatment. Clin Orthop Relat Res. (1982) 165:68–82. 7042168

[42] Mei R Qin W Zheng Y Wan Z Liu L. Role of adipose tissue derived exosomes in metabolic disease. Front Endocrinol. (2022) 13:873865. doi: 10.3389/fendo.2022.873865, PMID: 35600580 PMC9114355

[43] Zhang Y Yu M Tian W. Physiological and pathological impact of exosomes of adipose tissue. Cell Prolif. (2016) 49:3–13. doi: 10.1111/cpr.12233, PMID: 26776755 PMC6496788

[44] Connolly KD Rees DA James PE. Role of adipocyte-derived extracellular vesicles in vascular inflammation. Free Radic Biol Med. (2021) 172:58–64. doi: 10.1016/j.freeradbiomed.2021.04.031, PMID: 34052345

[45] Wadey RM Connolly KD Mathew D Walters G Rees DA James PE. Inflammatory adipocyte-derived extracellular vesicles promote leukocyte attachment to vascular endothelial cells. Atherosclerosis. (2019) 283:19–27. doi: 10.1016/j.atherosclerosis.2019.01.013, PMID: 30771557

[46] Zhang Y Bi J Huang J Tang Y Du S Li P. Exosome: a review of its classification, isolation techniques, storage, diagnostic and targeted therapy applications. Int J Nanomedicine. (2020) 15:6917–34. doi: 10.2147/IJN.S264498, PMID: 33061359 PMC7519827

[47] Ruan X Zhao W. Brown adipocyte-derived exosomes in type 2 diabetes mellitus impair endothelial function via regulating intracellular calcium cycle. Front Cardiovasc Med. (2025) 12:1546325. doi: 10.3389/fcvm.2025.1546325, PMID: 40416809 PMC12098568

[48] Ying W Riopel M Bandyopadhyay G Dong Y Birmingham A Seo JB. Adipose tissue macrophage-derived Exosomal miRNAs can modulate *in vivo* and *in vitro* insulin sensitivity. Cell. (2017) 171:372–384.e12. doi: 10.1016/j.cell.2017.08.035, PMID: 28942920

[49] Xu M Ji J Jin D Wu Y Wu T Lin R. The biogenesis and secretion of exosomes and multivesicular bodies (MVBs): intercellular shuttles and implications in human diseases. Genes Dis. (2023) 10:1894–907. doi: 10.1016/j.gendis.2022.03.021, PMID: 37492712 PMC10363595

[50] Liu Y Wang C Wei M Yang G Yuan L. Multifaceted roles of adipose tissue-derived exosomes in physiological and pathological conditions. Front Physiol. (2021) 12:669429. doi: 10.3389/fphys.2021.669429, PMID: 33959041 PMC8093393

[51] Suganami T Nishida J Ogawa Y. A paracrine loop between adipocytes and macrophages aggravates inflammatory changes: role of free fatty acids and tumor necrosis factor alpha. Arterioscler Thromb Vasc Biol. (2005) 25:2062–8. doi: 10.1161/01.ATV.0000183883.72263.13, PMID: 16123319

[52] Ouchi N Parker JL Lugus JJ Walsh K. Adipokines in inflammation and metabolic disease. Nat Rev Immunol. (2011) 11:85–97. doi: 10.1038/nri2921, PMID: 21252989 PMC3518031

[53] Cybulsky MI Iiyama K Li H Zhu S Chen M Iiyama M. A major role for VCAM-1, but not ICAM-1, in early atherosclerosis. J Clin Invest. (2001) 107:1255–62. doi: 10.1172/JCI11871, PMID: 11375415 PMC209298

[54] Hulsmans M Holvoet P. MicroRNA-containing microvesicles regulating inflammation in association with atherosclerotic disease. Cardiovasc Res. (2013) 100:7–18. doi: 10.1093/cvr/cvt161, PMID: 23774505

[55] Zhao H Shang Q Pan Z Bai Y Li Z Zhang H. Exosomes from adipose-derived stem cells attenuate adipose inflammation and obesity through polarizing M2 macrophages and Beiging in white adipose tissue. Diabetes. (2018) 67:235–47. doi: 10.2337/db17-0356, PMID: 29133512

[56] Yamashita Y Morimoto T Kimura T. Venous thromboembolism: recent advancement and future perspective. J Cardiol. (2022) 79:79–89. doi: 10.1016/j.jjcc.2021.08.026, PMID: 34518074

[57] Moore EE Moore HB Kornblith LZ Neal MD Hoffman M Mutch NJ. Trauma-induced coagulopathy. Nat Rev Dis Primers. (2021) 7:30. doi: 10.1038/s41572-021-00264-3, PMID: 33927200 PMC9107773

[58] Fröhlich M Mutschler M Caspers M Nienaber U Jäcker V Driessen A. Trauma-induced coagulopathy upon emergency room arrival: still a significant problem despite increased awareness and management? Eur J Trauma Emerg Surg. (2019) 45:115–24. doi: 10.1007/s00068-017-0884-5, PMID: 29170791

[59] Hayakawa M. Pathophysiology of trauma-induced coagulopathy: disseminated intravascular coagulation with the fibrinolytic phenotype. J Intensive Care. (2017) 5:14. doi: 10.1186/s40560-016-0200-1, PMID: 28289544 PMC5282695

[60] Maegele M Schöchl H Menovsky T Maréchal H Marklund N Buki A. Coagulopathy and haemorrhagic progression in traumatic brain injury: advances in mechanisms, diagnosis, and management. Lancet Neurol. (2017) 16:630–47. doi: 10.1016/S1474-4422(17)30197-7, PMID: 28721927

[61] Zhang J Zhang F Dong JF. Coagulopathy induced by traumatic brain injury: systemic manifestation of a localized injury. Blood. (2018) 131:2001–6. doi: 10.1182/blood-2017-11-784108, PMID: 29507078 PMC5934798

[62] Dyer MR Alexander W Hassoune A Chen Q Brzoska T Alvikas J. Platelet-derived extracellular vesicles released after trauma promote hemostasis and contribute to DVT in mice. J Thromb Haemost. (2019) 17:1733–45. doi: 10.1111/jth.14563, PMID: 31294514 PMC6773503

[63] Melki I Tessandier N Zufferey A Boilard E. Platelet microvesicles in health and disease. Platelets. (2017) 28:214–21. doi: 10.1080/09537104.2016.1265924, PMID: 28102737

[64] Zwaal RF Comfurius P Bevers EM. Surface exposure of phosphatidylserine in pathological cells. Cell Mol Life Sci. (2005) 62:971–88. doi: 10.1007/s00018-005-4527-3, PMID: 15761668 PMC11924510

[65] Tripisciano C Weiss R Eichhorn T Spittler A Heuser T Fischer MB. Different potential of extracellular vesicles to support thrombin generation: contributions of phosphatidylserine, tissue factor, and cellular origin. Sci Rep. (2017) 7:6522. doi: 10.1038/s41598-017-03262-2, PMID: 28747771 PMC5529579

[66] Giesen PL Rauch U Bohrmann B Kling D Roqué M Fallon JT. Blood-borne tissue factor: another view of thrombosis. Proc Natl Acad Sci USA. (1999) 96:2311–5. doi: 10.1073/pnas.96.5.2311, PMID: 10051638 PMC26780

[67] Brambilla M Camera M Colnago D Marenzi G De Metrio M Giesen PL. Tissue factor in patients with acute coronary syndromes: expression in platelets, leukocytes, and platelet-leukocyte aggregates. Arterioscler Thromb Vasc Biol. (2008) 28:947–53. doi: 10.1161/ATVBAHA.107.161471, PMID: 18292391

[68] Gidlöf O van der Brug M Ohman J Gilje P Olde B Wahlestedt C. Platelets activated during myocardial infarction release functional miRNA, which can be taken up by endothelial cells and regulate ICAM1 expression. Blood. (2013) 121:3908–3917, S3901–3926. doi: 10.1182/blood-2012-10-461798 23493781

[69] Brinkmann V Reichard U Goosmann C Fauler B Uhlemann Y Weiss DS. Neutrophil extracellular traps kill bacteria. Science (New York, NY). (2004) 303:1532–5. doi: 10.1126/science.1092385, PMID: 15001782

[70] Fuchs TA Brill A Duerschmied D Schatzberg D Monestier M Myers DD Jr. Extracellular DNA traps promote thrombosis. Proc Natl Acad Sci USA. (2010) 107:15880–5. doi: 10.1073/pnas.1005743107, PMID: 20798043 PMC2936604

[71] Vulliamy P Gillespie S Armstrong PC Allan HE Warner TD Brohi K. Histone H4 induces platelet ballooning and microparticle release during trauma hemorrhage. Proc Natl Acad Sci USA. (2019) 116:17444–9. doi: 10.1073/pnas.1904978116, PMID: 31405966 PMC6717295

[72] Martinod K Demers M Fuchs TA Wong SL Brill A Gallant M. Neutrophil histone modification by peptidylarginine deiminase 4 is critical for deep vein thrombosis in mice. Proc Natl Acad Sci USA. (2013) 110:8674–9. doi: 10.1073/pnas.1301059110, PMID: 23650392 PMC3666755

[73] Massberg S Grahl L von Bruehl ML Manukyan D Pfeiler S Goosmann C. Reciprocal coupling of coagulation and innate immunity via neutrophil serine proteases. Nat Med. (2010) 16:887–96. doi: 10.1038/nm.2184, PMID: 20676107

[74] Moore HB Moore EE Lawson PJ Gonzalez E Fragoso M Morton AP. Fibrinolysis shutdown phenotype masks changes in rodent coagulation in tissue injury versus hemorrhagic shock. Surgery. (2015) 158:386–92. doi: 10.1016/j.surg.2015.04.008, PMID: 25979440 PMC4492895

[75] von Brühl ML Stark K Steinhart A Chandraratne S Konrad I Lorenz M. Monocytes, neutrophils, and platelets cooperate to initiate and propagate venous thrombosis in mice *in vivo*. J Exp Med. (2012) 209:819–35. doi: 10.1084/jem.20112322, PMID: 22451716 PMC3328366

[76] Matijevic N Wang YW Wade CE Holcomb JB Cotton BA Schreiber MA. Cellular microparticle and thrombogram phenotypes in the prospective observational multicenter major trauma transfusion (PROMMTT) study: correlation with coagulopathy. Thromb Res. (2014) 134:652–8. doi: 10.1016/j.thromres.2014.07.023, PMID: 25086657 PMC4160305

[77] Claes L Recknagel S Ignatius A. Fracture healing under healthy and inflammatory conditions. Nat Rev Rheumatol. (2012) 8:133–43. doi: 10.1038/nrrheum.2012.1, PMID: 22293759

[78] Marsell R Einhorn TA. The biology of fracture healing. Injury. (2011) 42:551–5. doi: 10.1016/j.injury.2011.03.031, PMID: 21489527 PMC3105171

[79] Qin Y Wang L Gao Z Chen G Zhang C. Bone marrow stromal/stem cell-derived extracellular vesicles regulate osteoblast activity and differentiation *in vitro* and promote bone regeneration *in vivo*. Sci Rep. (2016) 6:21961. doi: 10.1038/srep21961, PMID: 26911789 PMC4766421

[80] Antonova E Le TK Burge R Mershon J. Tibia shaft fractures: costly burden of nonunions. BMC Musculoskelet Disord. (2013) 14:42. doi: 10.1186/1471-2474-14-42, PMID: 23351958 PMC3573940

[81] Meng F Xue X Yin Z Gao F Wang X Geng Z. Research Progress of exosomes in bone diseases: mechanism, diagnosis and therapy. Front Bioeng Biotechnol. (2022) 10:866627. doi: 10.3389/fbioe.2022.866627, PMID: 35497358 PMC9039039

[82] Zhang L Jiao G Ren S Zhang X Li C Wu W. Exosomes from bone marrow mesenchymal stem cells enhance fracture healing through the promotion of osteogenesis and angiogenesis in a rat model of nonunion. Stem Cell Res Ther. (2020) 11:38. doi: 10.1186/s13287-020-1562-9, PMID: 31992369 PMC6986095

[83] Kim WJ Shin HL Kim BS Kim HJ Ryoo HM. RUNX2-modifying enzymes: therapeutic targets for bone diseases. Exp Mol Med. (2020) 52:1178–84. doi: 10.1038/s12276-020-0471-4, PMID: 32788656 PMC8080656

[84] Jiang Y Zhang J Li Z Jia G. Bone marrow mesenchymal stem cell-derived Exosomal miR-25 regulates the ubiquitination and degradation of Runx2 by SMURF1 to promote fracture healing in mice. Front Med. (2020) 7:577578. doi: 10.3389/fmed.2020.577578, PMID: 33425934 PMC7793965

[85] Chen H Ji X She F Gao Y Tang P. miR-628-3p regulates osteoblast differentiation by targeting RUNX2: possible role in atrophic non-union. Int J Mol Med. (2017) 39:279–86. doi: 10.3892/ijmm.2016.2839, PMID: 28035362 PMC5358698

[86] Peng H Lu SL Bai Y Fang X Huang H Zhuang XQ. MiR-133a inhibits fracture healing via targeting RUNX2/BMP2. Eur Rev Med Pharmacol Sci. (2018) 22:2519–26. doi: 10.26355/eurrev_201805_14914, PMID: 29771401

[87] Zhang S Lu C Zheng S Hong G. Hydrogel loaded with bone marrow stromal cell-derived exosomes promotes bone regeneration by inhibiting inflammatory responses and angiogenesis. World J Stem Cells. (2024) 16:499–511. doi: 10.4252/wjsc.v16.i5.499, PMID: 38817325 PMC11135248

[88] Liu W Li L Rong Y Qian D Chen J Zhou Z. Hypoxic mesenchymal stem cell-derived exosomes promote bone fracture healing by the transfer of miR-126. Acta Biomater. (2020) 103:196–212. doi: 10.1016/j.actbio.2019.12.020, PMID: 31857259

[89] Lu GD Cheng P Liu T Wang Z. BMSC-derived Exosomal miR-29a promotes angiogenesis and osteogenesis. Front Cell Dev Biol. (2020) 8:608521. doi: 10.3389/fcell.2020.608521, PMID: 33363169 PMC7755650

[90] Sims NA Martin TJ. Osteoclasts provide coupling signals to osteoblast lineage cells through multiple mechanisms. Annu Rev Physiol. (2020) 82:507–29. doi: 10.1146/annurev-physiol-021119-034425, PMID: 31553686

[91] Boyce BF Xing L. Functions of RANKL/RANK/OPG in bone modeling and remodeling. Arch Biochem Biophys. (2008) 473:139–46. doi: 10.1016/j.abb.2008.03.018, PMID: 18395508 PMC2413418

[92] Holliday LS Patel SS Rody WJ Jr. RANKL and RANK in extracellular vesicles: surprising new players in bone remodeling. Extracell Vesicles Circ Nucl Acids. (2021) 2:18–28. doi: 10.20517/evcna.2020.02, PMID: 33982033 PMC8112638

[93] McDonald MM Khoo WH Ng PY Xiao Y Zamerli J Thatcher P. Osteoclasts recycle via osteomorphs during RANKL-stimulated bone resorption. Cell. (2021) 184:1330–1347.e1313. doi: 10.1016/j.cell.2021.02.002 33636130 PMC7938889

[94] Sun P Wang M Yin GY. Endogenous parathyroid hormone (PTH) signals through osteoblasts via RANKL during fracture healing to affect osteoclasts. Biochem Biophys Res Commun. (2020) 525:850–6. doi: 10.1016/j.bbrc.2020.02.177, PMID: 32169280

[95] Furuta T Miyaki S Ishitobi H Ogura T Kato Y Kamei N. Mesenchymal stem cell-derived exosomes promote fracture healing in a mouse model. Stem Cells Transl Med. (2016) 5:1620–30. doi: 10.5966/sctm.2015-0285, PMID: 27460850 PMC5189643

[96] Yu C Chen L Zhou W Hu L Xie X Lin Z. Injectable Bacteria-sensitive hydrogel promotes repair of infected fractures via sustained release of miRNA antagonist. ACS Appl Mater Interfaces. (2022) 14:34427–42. doi: 10.1021/acsami.2c08491, PMID: 35866896 PMC9354009

[97] Zhang Y Hao Z Wang P Xia Y Wu J Xia D. Exosomes from human umbilical cord mesenchymal stem cells enhance fracture healing through HIF-1α-mediated promotion of angiogenesis in a rat model of stabilized fracture. Cell Prolif. (2019) 52:e12570. doi: 10.1111/cpr.12570, PMID: 30663158 PMC6496165

[98] Jin S Luo Z Cai Y Wen J Lu P Fu X. Exosome-functionalized heterogeneous nanofibrous scaffolds repair bone defects accompanied by muscle injury. Chem Eng J. (2024) 485:149681. doi: 10.1016/j.cej.2024.149681

[99] Huang H Xiao L Fang L Lei M Liu Z Gao S. Static topographical Cue combined with dynamic fluid stimulation enhances the macrophage extracellular vesicle yield and therapeutic potential for bone defects. ACS Nano. (2025) 19:8667–91. doi: 10.1021/acsnano.4c15201, PMID: 39998493

[100] Gardiner C Di Vizio D Sahoo S Théry C Witwer KW Wauben M. Techniques used for the isolation and characterization of extracellular vesicles: results of a worldwide survey. J Extracell Vesicles. (2016) 5:32945. doi: 10.3402/jev.v5.32945, PMID: 27802845 PMC5090131

[101] Welsh JA Goberdhan DCI O'Driscoll L Buzas EI Blenkiron C Bussolati B. Minimal information for studies of extracellular vesicles (MISEV2023): from basic to advanced approaches. J Extracell Vesicles. (2024) 13:e12404. doi: 10.1002/jev2.12404, PMID: 38326288 PMC10850029

[102] Visan KS Lobb RJ Ham S Lima LG Palma C Edna CPZ. Comparative analysis of tangential flow filtration and ultracentrifugation, both combined with subsequent size exclusion chromatography, for the isolation of small extracellular vesicles. J Extracell Vesicles. (2022) 11:e12266. doi: 10.1002/jev2.12266, PMID: 36124834 PMC9486818

[103] Dixson AC Dawson TR Di Vizio D Weaver AM. Context-specific regulation of extracellular vesicle biogenesis and cargo selection. Nat Rev Mol Cell Biol. (2023) 24:454–76. doi: 10.1038/s41580-023-00576-0, PMID: 36765164 PMC10330318

[104] Kobayashi H Shiba T Yoshida T Bolidong D Kato K Sato Y. Precise analysis of single small extracellular vesicles using flow cytometry. Sci Rep. (2024) 14:7465. doi: 10.1038/s41598-024-57974-3, PMID: 38553534 PMC10980769

[105] Görgens A Bremer M Ferrer-Tur R Murke F Tertel T Horn PA. Optimisation of imaging flow cytometry for the analysis of single extracellular vesicles by using fluorescence-tagged vesicles as biological reference material. J Extracell Vesicles. (2019) 8:1587567. doi: 10.1080/20013078.2019.1587567, PMID: 30949308 PMC6442110

[106] Welsh JA Van Der Pol E Arkesteijn GJA Bremer M Brisson A Coumans F. MIFlowCyt-EV: a framework for standardized reporting of extracellular vesicle flow cytometry experiments. J Extracell Vesicles. (2020) 9:1713526. doi: 10.1080/20013078.2020.1713526, PMID: 32128070 PMC7034442

[107] Haraszti RA Didiot MC Sapp E Leszyk J Shaffer SA Rockwell HE. High-resolution proteomic and lipidomic analysis of exosomes and microvesicles from different cell sources. J Extracell Vesicles. (2016) 5:32570. doi: 10.3402/jev.v5.32570, PMID: 27863537 PMC5116062

[108] Choi DS Kim DK Kim YK Gho YS. Proteomics, transcriptomics and lipidomics of exosomes and ectosomes. Proteomics. (2013) 13:1554–71. doi: 10.1002/pmic.201200329, PMID: 23401200

[109] Lam SM Zhang C Wang Z Ni Z Zhang S Yang S. A multi-omics investigation of the composition and function of extracellular vesicles along the temporal trajectory of COVID-19. Nat Metab. (2021) 3:909–22. doi: 10.1038/s42255-021-00425-4, PMID: 34158670

[110] Wang ZG He ZY Liang S Yang Q Cheng P Chen AM. Comprehensive proteomic analysis of exosomes derived from human bone marrow, adipose tissue, and umbilical cord mesenchymal stem cells. Stem Cell Res Ther. (2020) 11:511. doi: 10.1186/s13287-020-02032-8, PMID: 33246507 PMC7694919

[111] Zhang Y Lan M Chen Y. Minimal information for studies of extracellular vesicles (MISEV): ten-year evolution (2014-2023). Pharmaceutics. (2024) 16:1394. doi: 10.3390/pharmaceutics16111394, PMID: 39598518 PMC11597804

[112] Chen YF Luh F Ho YS Yen Y. Exosomes: a review of biologic function, diagnostic and targeted therapy applications, and clinical trials. J Biomed Sci. (2024) 31:67. doi: 10.1186/s12929-024-01055-0, PMID: 38992695 PMC11238361

[113] Alvarez-Erviti L Seow Y Yin H Betts C Lakhal S Wood MJ. Delivery of siRNA to the mouse brain by systemic injection of targeted exosomes. Nat Biotechnol. (2011) 29:341–5. doi: 10.1038/nbt.1807, PMID: 21423189

[114] El Andaloussi S Mäger I Breakefield XO Wood MJ. Extracellular vesicles: biology and emerging therapeutic opportunities. Nat Rev Drug Discov. (2013) 12:347–57. doi: 10.1038/nrd3978 23584393

[115] Loconte L Arguedas D El R Zhou A Chipont A Guyonnet L. Detection of the interactions of tumour derived extracellular vesicles with immune cells is dependent on EV-labelling methods. J Extracell Vesicles. (2023) 12:e12384. doi: 10.1002/jev2.12384, PMID: 38031976 PMC10687762

[116] Gangadaran P Hong CM Ahn BC. An update on *in vivo* imaging of extracellular vesicles as drug delivery vehicles. Front Pharmacol. (2018) 9:169. doi: 10.3389/fphar.2018.00169, PMID: 29541030 PMC5835830

[117] Hwang DW Choi H Jang SC Yoo MY Park JY Choi NE. Noninvasive imaging of radiolabeled exosome-mimetic nanovesicle using (99m)Tc-HMPAO. Sci Rep. (2015) 5:15636. doi: 10.1038/srep15636, PMID: 26497063 PMC4620485

[118] Choi H Kim MY Kim DH Yun H Oh BK Kim SB. Quantitative biodistribution and pharmacokinetics study of GMP-grade exosomes labeled with (89)Zr radioisotope in mice and rats. Pharmaceutics. (2022) 14:1118. doi: 10.3390/pharmaceutics14061118, PMID: 35745690 PMC9229812

[119] Hikita T Miyata M Watanabe R Oneyama C. *In vivo* imaging of long-term accumulation of cancer-derived exosomes using a BRET-based reporter. Sci Rep. (2020) 10:16616. doi: 10.1038/s41598-020-73580-5, PMID: 33024173 PMC7538576

[120] Gupta D Liang X Pavlova S Wiklander OPB Corso G Zhao Y. Quantification of extracellular vesicles *in vitro* and *in vivo* using sensitive bioluminescence imaging. J Extracell Vesicles. (2020) 9:1800222. doi: 10.1080/20013078.2020.1800222, PMID: 32944187 PMC7481830

[121] Li B Kugeratski FG Kalluri R. A novel machine learning algorithm selects proteome signature to specifically identify cancer exosomes. eLife. (2024) 12:RP90390. doi: 10.7554/eLife.90390, PMID: 38529947 PMC10965221

[122] Lucien F Gustafson D Lenassi M Li B Teske JJ Boilard E. MIBlood-EV: minimal information to enhance the quality and reproducibility of blood extracellular vesicle research. J Extracell Vesicles. (2023) 12:e12385. doi: 10.1002/jev2.12385, PMID: 38063210 PMC10704543

